# Activity-dependent ectopic action potentials in regular-spiking neurons of the neocortex

**DOI:** 10.3389/fncel.2023.1267687

**Published:** 2023-11-09

**Authors:** Yizhen Z. Zhang, Stella Sapantzi, Alice Lin, Savannah R. Doelfel, Barry W. Connors, Brian B. Theyel

**Affiliations:** ^1^Department of Neuroscience, Brown University, Providence, RI, United States; ^2^National Institute of Dental and Craniofacial Research, National Institutes of Health, Bethesda, MD, United States; ^3^Department of Psychiatry and Human Behavior, Brown University, Providence, RI, United States

**Keywords:** ectopic, action potential, regular spiking cell, neocortex, pyramidal neuron, spike, backpropagating action potential

## Abstract

**Introduction:**

Action potentials usually travel orthodromically along a neuron’s axon, from the axon initial segment (AIS) toward the presynaptic terminals. Under some circumstances action potentials also travel in the opposite direction, antidromically, after being initiated at a distal location. Given their initiation at an atypical site, we refer to these events as “ectopic action potentials.” Ectopic action potentials (EAPs) were initially observed in pathological conditions including seizures and nerve injury. Several studies have described regular-spiking (RS) pyramidal neurons firing EAPs in seizure models. Under nonpathological conditions, EAPs were reported in a few populations of neurons, and our group has found that EAPs can be induced in a large proportion of parvalbumin-expressing interneurons in the neocortex. Nevertheless, to our knowledge there have been no prior reports of ectopic firing in the largest population of neurons in the neocortex, pyramidal neurons, under nonpathological conditions.

**Methods:**

We performed in vitro recordings utilizing the whole-cell patch clamp technique. To elicit EAPs, we triggered orthodromic action potentialswith either long, progressively increasing current steps, or with trains of brief pulses at 30, 60, or 100 Hz delivered in 3 different ways, varying in stimulus and resting period duration.

**Results:**

We found that a large proportion (72.7%) of neocortical RS cells from mice can fire EAPs after a specific stimulus in vitro, and that most RS cells (56.1%) are capable of firing EAPs across a broad range of stimulus conditions. Of the 37 RS neurons in which we were able to elicit EAPs, it took an average of 863.8 orthodromic action potentials delivered over the course of an average of ~81.4 s before the first EAP was seen. We observed that some cells responded to specific stimulus frequencies while less selective, suggesting frequency tuning in a subset of the cells.

**Discussion:**

Our findings suggest that pyramidal cells can integrate information over long time-scales before briefly entering a mode of self-generated firing that originates in distal axons. The surprising ubiquity of EAP generation in RS cells raises interesting questions about the potential roles of ectopic spiking in information processing, cortical oscillations, and seizure susceptibility.

## 1 Introduction

The properties and mechanisms of action potentials in mammalian neurons are largely understood. In most cells the action potential originates at the axon initial segment (AIS), near the cell body, and then travels orthodromically down the axon to the terminals, as well as antidromically into the cell body and dendrites. For the purposes of this article, we will refer to these action potentials as “axon initial segment action potentials” (AIS-APs). Under certain conditions, neurons are also capable of producing action potentials that initiate distally in the axon, its branches, or its terminals. These action potentials, henceforth referred to as “ectopic action potentials” (EAPs), can travel antidromically toward the cell body as well as orthodromically through any axons and terminals distal to branch points the EAPs encounter enroute to the cell body (Gutnick and Prince, 1972, 1975; Pinault, 1995). EAPs in mammals have predominantly been recorded under pathological conditions, particularly in models of epilepsy (Gutnick and Prince, 1972, 1975; Pinault and Pumain, 1985; Pinault, 1995; Keros and Hablitz, 2005). EAPs have also been detected in injured cells after nerve trauma, where they may contribute to neuropathic pain (Amir et al., 2005; Devor, 2009). More recently, EAPs have also been occasionally reported in nonpathological animal models (Pinault, 1995; Sheffield et al., 2011).

Although most early reports of EAPs in the mammalian nervous system involved excitatory projection neurons in pathological tissue (Pinault, 1995), recent studies have shown that inhibitory interneurons can also generate EAPs. Sheffield et al. (2011) characterized persistent firing of EAPs, which they refer to as “barrages,” in 79% of inhibitory neuropeptide Y-expressing interneurons in the hippocampus; barrages arose after sufficient somatic stimulation in nonpathological tissue. These EAPs appear to initiate in the cells’ distal axons as a result of prolonged (several minutes) integration of AIS-APs (Sheffield et al., 2011; Elgueta et al., 2015; Deemyad et al., 2018). Suzuki et al. (2014) examined interneurons of the olfactory cortex that are capable of generating similar EAP barrages; these EAPs trigger inhibitory synaptic events in nearby pyramidal neurons. While Suzuki et al. (2014) found that 23% of fast-spiking cells (presumed to be parvalbumin-expressing interneurons) fired barrages of EAPs, our lab has found that a large percentage (~80%) of identified PV+ interneurons in both the primary somatosensory and orbitofrontal cortices fire EAPs (unpublished observations).

While it has been shown that neurons are capable of evoking EAPs under pathological conditions and in healthy inhibitory neocortical cells, as well as in hippocampal pyramidal cells (Papatheodoropoulos, 2008; Bähner et al., 2011; Dugladze et al., 2012; Thome et al., 2018), there have been no previous reports of widespread ectopic firing in healthy, excitatory pyramidal cells of the neocortex. Regular-spiking (RS) neurons are one of the most common physiological types of cells in the neocortex (Mountcastle et al., 1969). They are characterized by distinctly shaped AIS-APs with half-amplitude durations ranging from 0.6 to 1.0 ms, adapting frequencies during trains of evoked AIS-APs, and a prolonged afterhyperpolarization (Connors et al., 1982; McCormick et al., 1985; Bean, 2007). Most RS neurons are excitatory pyramidal neurons (McCormick et al., 1985; Connors and Gutnick, 1990). Pyramidal cells are among the most commonly studied neocortical neurons (Spruston, 2008), and it is possible that ectopic firing was either ignored or missed because experimental conditions were not sufficient to induce it. For instance, the commonly used artificial cerebrospinal fluid calcium concentration of 2.0 mM could be decreasing cell excitability, which may inhibit EAP generation compared to the physiological 1.2 mM concentration (Lu et al., 2010; Forsberg et al., 2019; Wang and Lu, 2023).

Here we examined whether RS neurons in nonpathological mouse neocortex can generate EAPs under a variety of stimulus conditions. We used whole-cell recording methods *in vitro,* and we applied a variety of stimulus protocols to evoke AIS-APs in layer 2/3 pyramidal cells of the mouse orbitofrontal cortex. We present data that suggest that the majority of these RS neurons are capable of firing EAPs, and that some are “frequency-tuned” to fire EAPs, with important implications for our understanding of neural processing. Such a phenomenon is likely to play a role in information processing, including being a form of feedforward excitation, and as a source of ‘random firing’ (Chao et al., 2005).

## 2 Results

**Figure 1:**
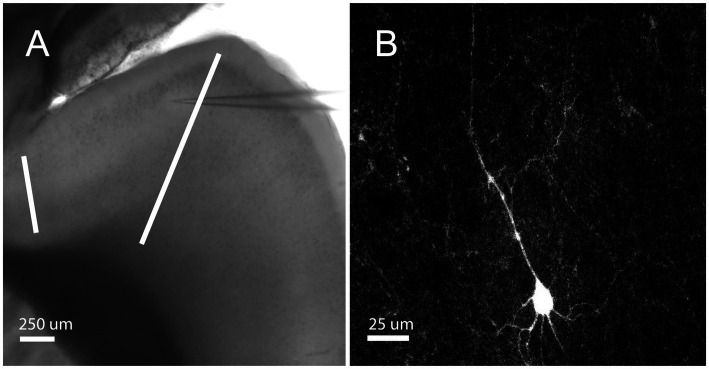
Orbitofrontal cortex location and RS Cell Example. **(A)** Mouse brain slice from an NTSR1 mouse under brightfield illumination with 4X objective. White bars enclosing the orbitofrontal cortex. Electrode patched onto RS cell in the orbitofrontal cortex. Scale bar: 250 μm. **(B)** Mouse brain slice from NTSR1 mouse with biocytin-filled cell from recording. Visualized on Olympus FV3000 confocal microscope with 60X objective lens. Scale bar: 25 μm.

Whole-cell somatic recordings were obtained from layer 2/3 neurons in sagittal slices of the orbitofrontal cortex (Figure 1A). Layer 2/3 was identified by its characteristic layer of cell bodies, which are readily apparent under brightfield illumination, that extends across the prefrontal cortex in sagittal slices. Recordings were physiologically identified and characterized by their distinct firing characteristics as RS cells (see Methods; McCormick et al., 1985; Connors and Gutnick, 1990). Here we report on 66 RS cells from 17 male and 14 female mice between the ages of postnatal day 18 and 60. Figure 1B depicts a sample RS pyramidal cell filled with biocytin.

### 2.1 Identification of the most effective method(s) to elicit EAPs

The stimulus conditions required to evoke ectopic firing in various cell types appear to vary. Elgueta et al. (2015), for instance, experimented with both a step-dependent stimulus protocol and specific frequency stimuli, including 30, 50, 75, or 100 Hz in their study of gyrus perisoma-inhibiting interneurons. They detected EAPs in 58–85% of these interneurons, whereas others determined that repeated current stimulus pulses efficiently evoked EAPs in their cells of interest (Sheffield et al., 2011).

**Figure 2:**
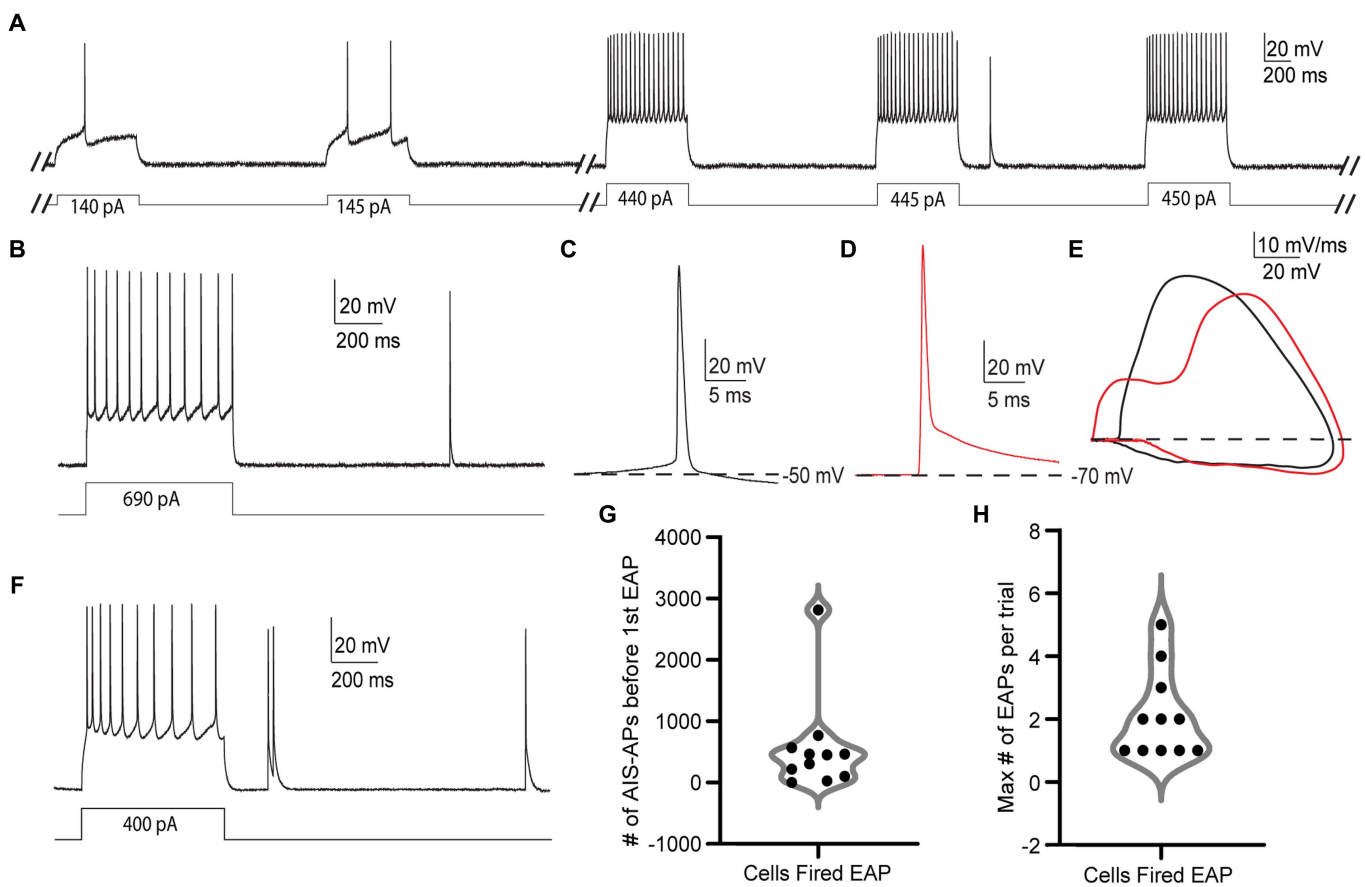
EAPs in RS cells are readily differentiated from AIS-APs, and they can be evoked by the step protocol. **(A)** Sample trials from the step protocol which consists of current steps starting at −25 pA and increasing by 5 pA every 3 s until depolarization block. Showing trials from the cell’s initial response to the current injection, and before, during, and after the first EAP. **(B)** Trial from the step protocol, during which a current injection of 690 pA elicits AIS-APs followed by a single EAP. **(C)** AIS-AP with the dotted line representing the membrane potential at −50 mV. **(D)** EAP rising from a membrane potential of −70 mV. **(E)** Phase plots (dV/dt vs. membrane potential) of the two different types of action potentials in **(A,B)**. AIS-APs are plotted in black and EAPs in red. **(F)** Trial from the step protocol showing AIS-APs evoked by a 400 pA current injection and followed by multiple EAPs. **(G)** Distribution of the number of AIS-APs preceding the first EAP in the 11 RS cells that fired EAPs. **(H)** The maximum number of EAPs fired in a single trial across the 11 recorded RS cells using the step protocol.

We first used 600 ms current stimulus pulses that sequentially increased in amplitude by 5 pA and were delivered repeatedly in 3 s long trials, hereafter referred to as the “step protocol” (Figure 2A). Upon reaching AIS-AP threshold potential as a result of the positive current injections, the neurons started firing trains of AIS-APs that gradually increased in frequency.

Out of the 66 cells tested under the step protocol, only 11 (16.7%) fired at least one EAP. The relatively small proportion of RS cells that fired EAPs during the step protocol differs from findings in hippocampal NPY cells (Sheffield et al., 2011) and parvalbumin positive interneurons (Suzuki et al., 2014, unpublished observations). We also noticed different numbers of EAPs being elicited in each trial depending on the cell, with some cells only eliciting one EAP in a trial (Figure 2B), and others firing multiple EAPs (Figure 2F). While detected in other cell types that fire EAPs, “barrages” of EAPs, defined as regular high-frequency trains of EAPs lasting at least 250 ms, were not present in any RS cell during the step protocol (Sheffield et al., 2013; Deemyad et al., 2018, unpublished observations).

### 2.2 Characterization of EAPs in RS neurons

The EAPs we observed in RS cells have characteristics that are distinct from AIS-APs, while they resemble EAPs described previously. Notably, an AIS-AP begins with a relatively long, slow depolarization until it reaches threshold (Figure 2C), whereas an EAP rises rapidly from the resting membrane potential without this preceding slow depolarization (Figure 2D). The swift rise from the resting membrane potential suggests that the action potential originated in the axon before propagating back into the soma. The EAPs that occurred also lacked the slow depolarization preceding AP threshold that is characteristic of AIS-APs. These features indicate initiation at a location electrotonically distant from the somatic recording site, with subsequent backpropagation into the cell body, and enable the experimenter to readily identify EAPs while recording. The differences between evoked orthodromic AIS-APs and EAPs as seen in somatic recordings are depicted in Figures 2A,B. Figure 2A depicts a trace of an AIS-AP evoked by a current step, and Figure 2B shows a trace of an EAP rising from resting membrane potential. The different trajectories of the two types of APs are most easily appreciated in phase plots of dV/dt as a function of membrane potential (Figure 2E). Note the higher peak dV/dt and AP amplitude for the EAP relative to the AIS-AP at membrane potentials below threshold. These findings are consistent with previously reported characteristics of EAPs (Gutnick and Prince, 1972; Gutnick and Prince, 1975; Pinault, 1995; Sheffield et al., 2011; Elgueta et al., 2015; Deemyad et al., 2018).

The EAP initiation threshold, defined as the total number of directly evoked AIS-APs required before the first EAP, was an average of 544 ± 788.2 and median of 305 AIS-APs (Figure 2G and Table 1). The integration of these AIS-APs before the first EAP typically occurred over the course of at least 1 min (mean of 319.64 ± 122.49 s; Table 1). The firing robustness, defined as the maximum number of EAPs fired in a single trial, was an average of 2.09 ± 1.38 (Figure 2H and Table 1).

**Table 1:** Quantification of initiation threshold, time to first EAP and robustness.

Protocol	Frequency	Initiation Threshold (# of AIS-APs before first EAP)	Time to first EAP (s)	Robustness (Max # of EAP per trial)
Step		544 ± 788.2 (305)	319.64 ± 122.49 (315)	2.09 ± 1.38 (2)
10 s	30 Hz	612 ± 394.4 (360)	34.00 ± 21.91 (20)	1.8 ± 1.79 (1)
	60 Hz	450 ± 372.29 (360)	25.00 ± 20.68 (20)	3.7 ± 3.12 (4)
	100 Hz	740 ± 919.78 (360)	40.00 ± 48.30 (25)	4.10 ± 4.01 (3.5)
3 s	30 Hz	315 ± 358.75 (195)	26.25 ± 35.88 (19.5)	1.38 ± 1.89 (4.5)
	60 Hz	270 ± 256.67 (150)	13.5 ± 12.83 (7.5)	7.25 ± 6.43 (6)
	100 Hz	483.33 ± 711.10 (150)	12.86 ± 14.50 (4.5)	6.5 ± 9.65 (2.5)
10 s without rest	30 Hz	2,880 ± 3054.70 (2880)	85.00 ± 63.64 (85)	43.5 ± 60.10 (43.5)
	60 Hz	2,340 ± 2811.69 (900)	130.0 ± 156.20 (50)	11.33 ± 9.07 (15)
	100 Hz	2,295 ± 1450.27 (2880)	127.5 ± 80.57 (16)	1.75 ± 0.96 (1.5)
Compiled		863.87 ± 1214.02 (360)	81.38 ± 57.7 (20)	5.69 ± 10.73 (3)

Mean ± standard deviation (median).

### 2.3 Specific frequency pulses frequently elicited EAPs

**Figure 3:**
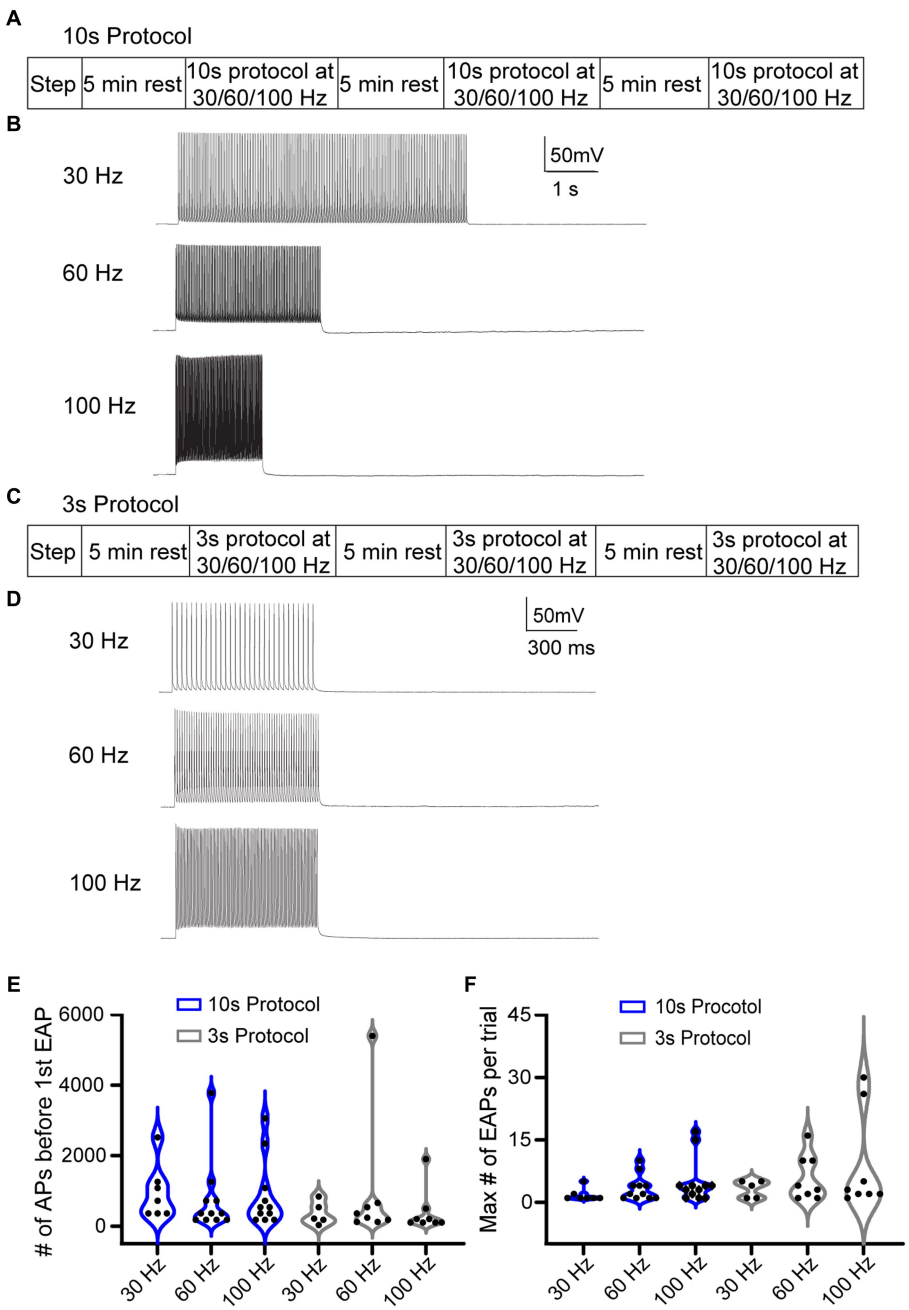
Different stimulation protocols can initiate EAPs. **(A)** 10 s protocol: sequence of the step protocol and three frequencies with a 5 min rest period in between. **(B)** Sample 10 s trials with 180 current pulses at 30, 60, and 100 Hz, respectively. **(C)** Similarly, for the 3 s protocol: sequence of step protocol and frequency stimulations with a 5 min rest in between. **(D)** Sample 3 s trials with 30, 60, 100 Hz current pulses, respectively, for 1 s. **(E)** Violin plot of the total number of AIS-APs present before the first EAP for the 30, 60, and 100 Hz stimuli with the 10s protocol (blue) and 3 s protocol (black). **(F)** Violin plot of the maximum number of EAPs per trial at the three different frequencies with the 10 s protocol (blue) and 3 s protocol (black). There was no statistically different number of AIS-APs before the first EAP or maximum number of EAPs per trial (*p* > 0.05; Kruskal–Wallis test).

Due to the low rate of EAP induction in RS cells using the step protocol, we explored multiple specific frequency pulse stimuli. We used three additional current injection protocols, which consisted of pulse trains eliciting AIS-APs at three different frequencies: 30, 60, and 100 Hz. To trigger AIS-APs at defined frequencies we used brief (2–3 ms), strong (2,500 ± 500 pA) stimulus pulses delivered at 30, 60, or 100 Hz. For each frequency, the current amplitude was titrated so that each pulse would elicit a single AIS-AP at least 80% of the time. We delivered current pulse stimuli in two ways: (1) either a fixed number of AIS-APs were elicited in each trial, or (2) AIS-APs were elicited at different frequencies for a shorter, fixed period. The first approach consisted of 10-s-long repeated trials, referred to as the “10 s protocol,” with 180 pulses delivered at 30, 60, or 100 Hz (Figures 3A,B) in each trial. Trials were repeated at each frequency until one of two conditions were met: (1) EAPs were elicited followed by at least 2 trials without EAPs, or (2) at least 4,000 AIS-APs were elicited. After this sequence, the cell was allowed to rest for 5 min before repeating it at a different frequency. All the frequencies were delivered to each cell in a random order. The second approach consisted of 3-s-long trials, referred to as the “3 s protocol,” with 1 s of current pulses at 30, 60, or 100 Hz (Figures 3C,D). Similarly, all the frequencies were delivered to each cell in a random order, similar to the 10s protocol described above. Since the stimulus time was consistent across frequencies here, the 30 Hz stimulation had 30 current pulses per trial in 1 s, the 60 Hz stimulation had 60 current pulses, and 100 Hz had 100 current pulses. In between applications of the frequency stimulations, we allowed the cell a rest period of 5 min. The 10 s protocol, in which the number of AIS-APs per trial was consistent across protocols, had a 72.7% (16/22) success rate for eliciting at least one EAP at one or more of the stimulus frequencies, while the 3 s protocol was effective only 57.1% (12/21) of the time (*p* = 0.14, Chi-squared test). We next compared the EAP initiation threshold and firing robustness between the two protocols. There were no statistically significant differences among the different frequencies from the two protocols (Figures 3E,F).

**Figure 4:**
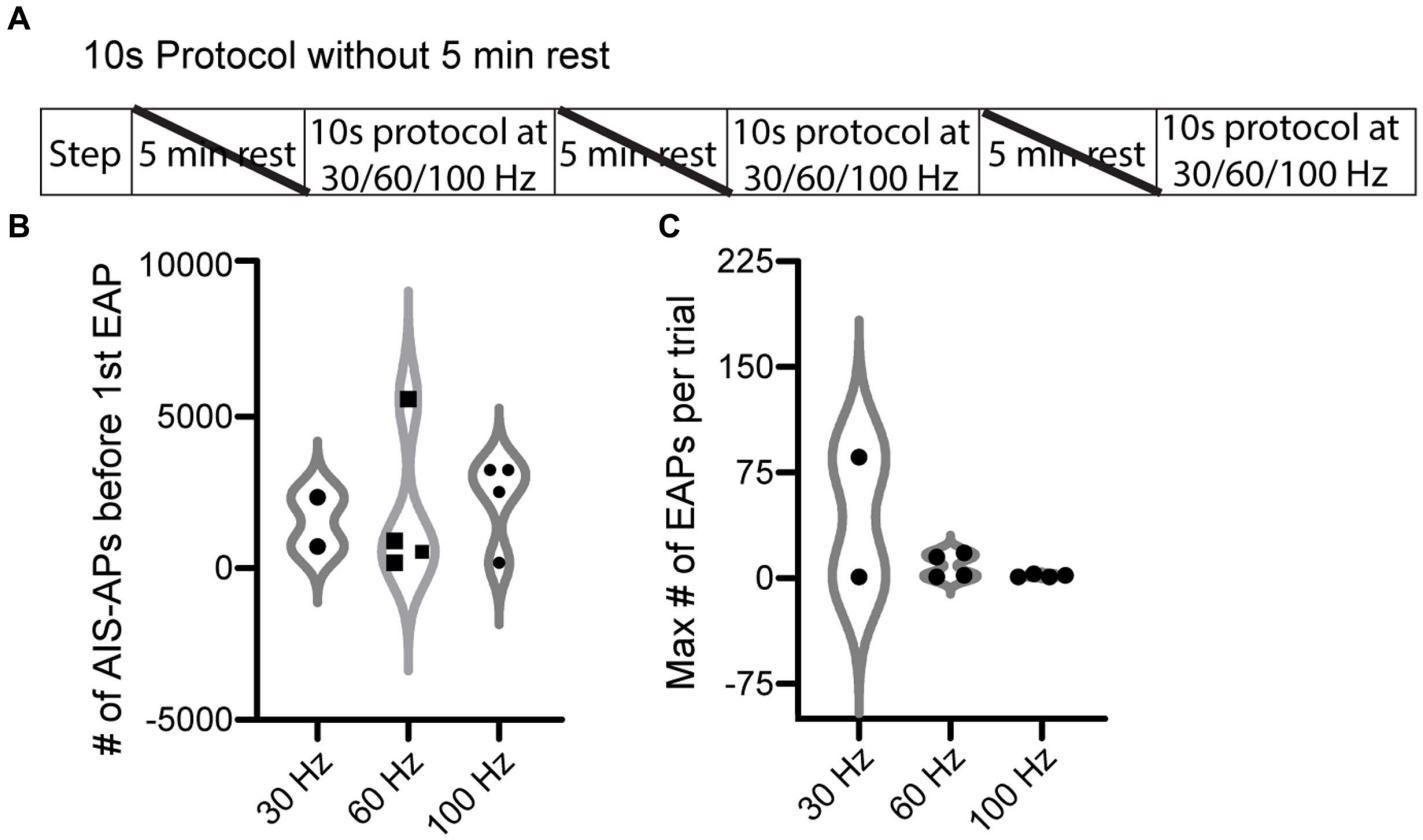
EAP threshold and robustness in response to 10 s protocol without rest periods. **(A)** ‘10 s without rest’ recording and stimulation timeline for each cell. Note that the 5 min rests between stimuli were not included here. **(B)** Violin plot of the 10 s protocol without rest at the three different frequencies of current pulses for initiation threshold (total number of AIS-APs present before the first EAP) (*p* > 0.05; Kruskal–Wallis test). **(C)** Violin plot of the EAP robustness (maximum number of EAPs per trial) at the three different frequencies (*p* > 0.05; Kruskal–Wallis test). For representative examples of cell firing patterns and rates during protocols please refer to Figure 3B. The only difference between the 10 s protocol and the 10 s without rest protocol is that a 5 min rest period was not included between protocols.

We next tested the hypothesis that AIS-AP accumulation at varying frequencies elicits EAPs more effectively compared to when the cell is allowed a rest period, which prevents AIS-AP accumulation. To do this, we removed the 5 min rest component from our most effective protocol, the 10 s (Figure 4A), immediately switching from one frequency to the next rather than allowing cells to reset in between protocols. We refer to this protocol as the ‘10 s without rest’ protocol. Contrary to our hypothesis that sequential, varying frequencies of stimulation would enhance EAP firing, removing the rest component dropped the effectiveness of initiating an EAP in the 10 s protocol from 72.7 to 39.1% (9/23) (*p* = 0.027, Chi-squared test). There were no statistically significant differences in the initiation threshold and firing robustness among the 30, 60, and 100 Hz (*p* > 0.05; Kruskal–Wallis test) (Figures 4B,C).

### 2.4 EAP threshold and robustness across protocols

**Figure 5:**
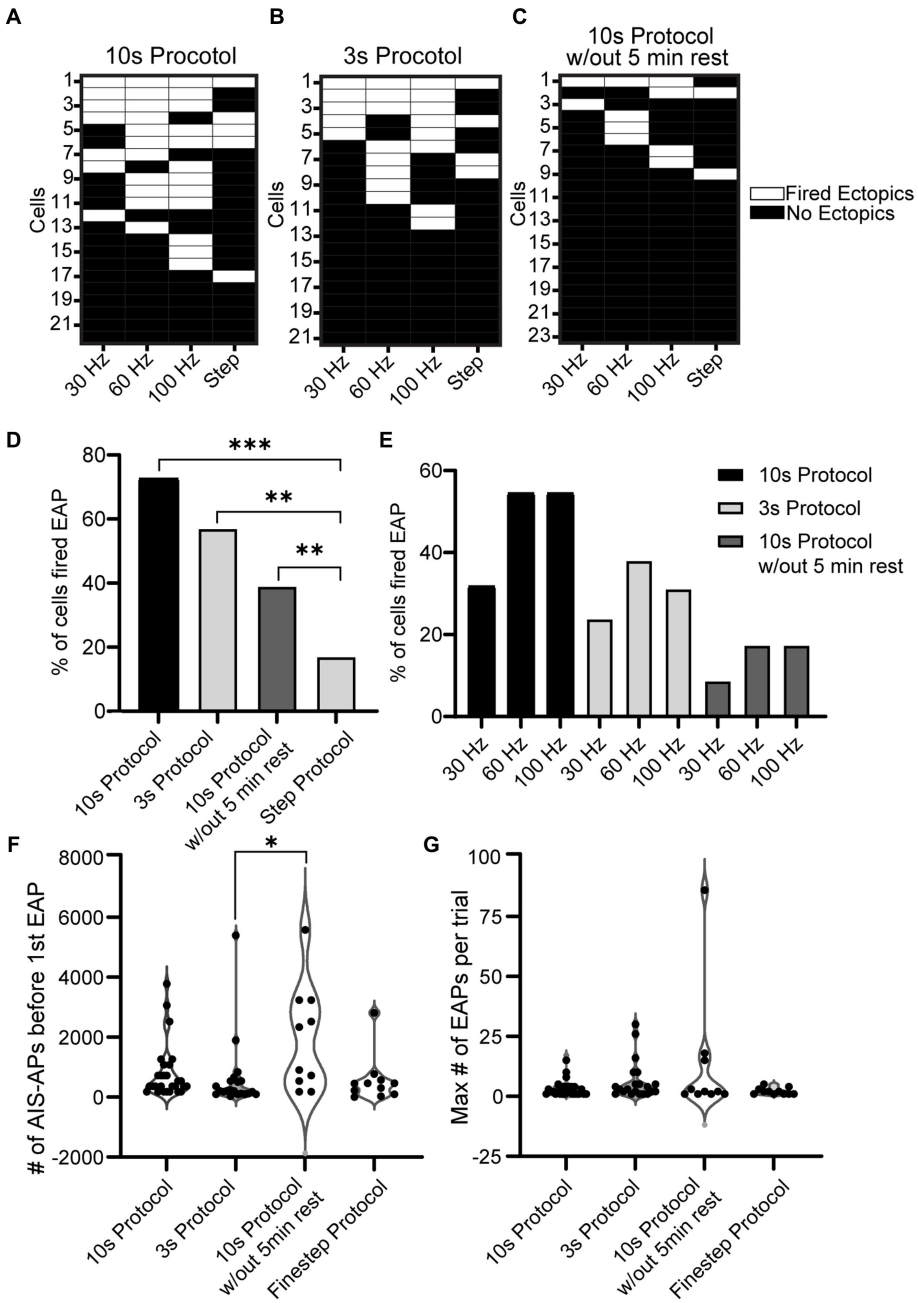
Comparison of EAP firing efficiency and robustness across all protocols. **(A−C)** Grid depicting three groups of cells for the three types of protocols used (10 s for **A**, 3 s for **B**, and 10 s without rest in **C**). Each cell is plotted in a row and the order is in descending order of EAP firing. If the cell shown in a row fired an EAP in response to a portion of the 10 s Protocol, the corresponding location is white, and if not the corresponding area is black. Note that 11/22 cells fired EAPs during multiple stimulation patterns in the 10 s protocol, while only 2/23 did in the 10 s protocol without rest. **(D)** The percentage of cells that fired EAP(s) for all the recorded cells from each protocol (step protocol and the three frequency protocols – 10 s protocol, 3 s protocol, 10 s protocol without 5 min rest). Upon comparison of the four protocols, there was a significant difference between the step protocol and the frequency stimulations in all three protocols (10, 3, and 10 s without rest with *p* = 7.9e-07, 2.5e-04, and 2.6e-02 respectively, Chi-squared test). There was no significant difference among the frequency protocols (*p* > 0.05, Chi-squared test). **(E)** Detailed comparison of the percentage of cells that fired EAPs in response to each of the three frequencies (30, 60, 100 Hz) for every frequency protocol. **(F)** Comparison of EAP initiation threshold (number of AIS-APs before the first EAP) among all four protocols. There was a significant difference between the threshold of the 10 s protocol and 3 s protocol (*p* = 0.008; Kruskal–Wallis test). **(G)** Comparison of EAP robustness among all four protocols. There was no significant difference among the robustness of the four protocols (*p* > 0.05; Kruskal–Wallis test).

To examine the overall EAP activity throughout the different stimuli, grids were made for the three protocols where each row is a cell and each column is a stimulus type (30, 60, 100 Hz, or step) (Figures 5A–C). If the cell was able to fire at least one EAP during that protocol/frequency combination, the area on the grid is white; if the cell did not fire any EAPs, the area on the grid is black. With the 10 s protocol, 16/22 of the cells fired EAPs. Of the cells that fired EAPs: 7/16 fired EAPs in response to 30 Hz stimulation, 11/16 fired EAPs in response to 60 Hz stimulation, and 12/16 fired EAPs in response to 100 Hz stimulation. Five cells fired EAPs in response to only one frequency protocol at: 30 Hz (1 cell), 60 Hz (1 cell), and 100 Hz (3 cells). Eight cells responded to two frequencies, and 3 cells fired EAPs in response to all three frequencies. Only one cell fired EAPs in response to only the step protocol (Figure 5A).

A similar plot was created for the 3 s protocol where 12/21 cells fired EAPs. Of the cells that fired EAPs: 5/12 fired EAPs in response to the 30 Hz protocol, 8/12 fired EAPs during the 60 Hz protocol, 8/12 fired EAPs in response to the 100 Hz protocol, and 4/12 fired EAPs in response to the step protocol. 4/12 cells only fired EAPs in response to a single frequency protocol: 60 Hz (2 cells), and 100 Hz (2 cells); 3 cells fired EAPs in response to two frequency protocols, and 3 cells fired EAPs in response to all frequency protocols (Figure 5B). For this and the previous 10s protocol, there seems to be a trend for RS cells that fired EAPs during one protocol to be more likely to fire EAPs in response to others as well.

Lastly a plot was created for the 10s protocol without rest where 9/23 cells fired EAPs in response to at least one protocol. Only two cells fired EAPs in response to the steps protocol. Six cells fired EAPs in response to a single frequency: 30 Hz (1 cell), 60 Hz (3 cells), and 100 Hz (2 cells). No cells fired EAPs in response to two frequency protocols, while one cell fired EAPs in response to all three frequency protocols (Figure 5C).

Upon analyzing the percent of cells that fired EAPs across all protocols, regardless of frequency, there was no significance among the 10 s protocol, 3 s protocol, and 10s protocol without 5 min rest. There was, however, a significant difference between these three protocols and the step protocol (*p* = 7.9e-07, 2.5e-04, and 2.6e-02 respectively, Chi-squared test) (Figure 5D). A more detailed graph was created to visualize the percent of cells that fired for each protocol and its respective frequency (Figure 5E). This shows that there is a trend indicating that the 30 Hz stimulus is less effective in eliciting EAPs compared to 60 or 100 Hz, regardless of the protocol.

A comparison of the initiation thresholds among all protocols shows that, among cells that fired EAPs, the 3 s protocol required the fewest AIS-APs to initiate an EAP at an average of 356.14 ± 442.17 and there was a significant difference between the threshold for the 3 s protocol and the 10s protocol without 5 min rest (*p* < 0.05; Kruskal-Wallis test) while there was no other significant difference among all other protocols (Figure 5F). We did not detect a significant difference in EAP firing robustness among the four protocols (*p* > 0.05; Kruskal–Wallis test) (Figure 5G).

To investigate the relationship between a cell’s ability to fire EAPs and its intrinsic properties, we examined the input resistance, firing threshold, and AIS-AP halfwidth for the cells in the most effective protocol (10 s protocol). We found no correlation between any of these properties and the probability that a cell fires EAPs (*p* > 0.05; *T*-test).

### 2.5 EAP amplitude variability

**Figure 6:**
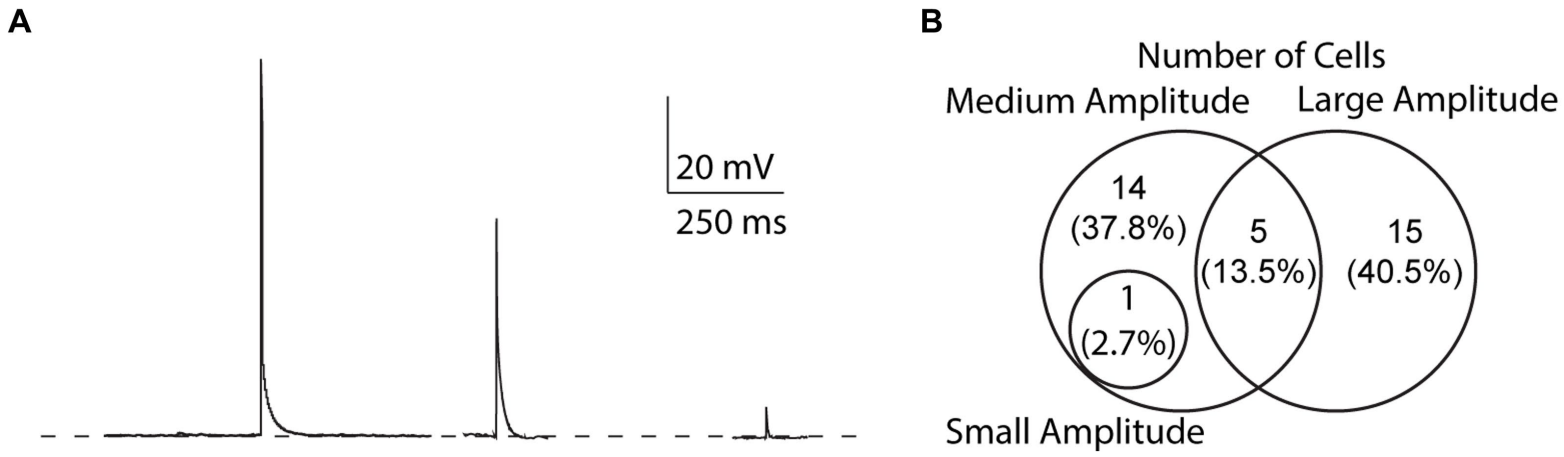
Variability of EAP amplitudes. **(A)** Compiled sample EAPs of varying amplitudes. Sample EAPs are categorized based on their full amplitudes as large (>80 mV), medium (10–80 mV), or small (<10 mV) amplitudes, respectively. **(B)** Across all protocols, 1 cell (2.7% of all cells) fired both small and medium amplitude EAPs, 14 (37.8%) fired only medium amplitude EAPs, 5 (13.5%) fired both medium and large amplitude EAPs, and 15 (40.5%) fired only large amplitude EAPs.

We found that the amplitude of EAPs can vary, similar to EAPs from other cell types (Sheffield et al., 2013; Suzuki et al., 2014; Elgueta et al., 2015; Deemyad et al., 2018, unpublished observations). We categorized EAPs based on their amplitude and assigned them into three groups: (1) “small” for EAPs with amplitudes smaller than 10 mV, (2) “medium” for EAP amplitudes between 10 and 80 mV, and (3) “large” for EAP amplitudes greater than 80 mV (Figure 6A). Out of 37 cells that fired EAPs, only one cell fired small EAPs (2.7%), while medium and large EAPs were observed in 20 cells each (54%) (Figure 6B). Most cells only fired EAPs in one single amplitude group (29/37), 6/37 cells fired EAPs in two different amplitude groups, while there were no cells that fired EAPs in all three amplitude groups (Figure 6B).

### 2.6 No correlation between EAP firing and sex or age

**Figure 7:**
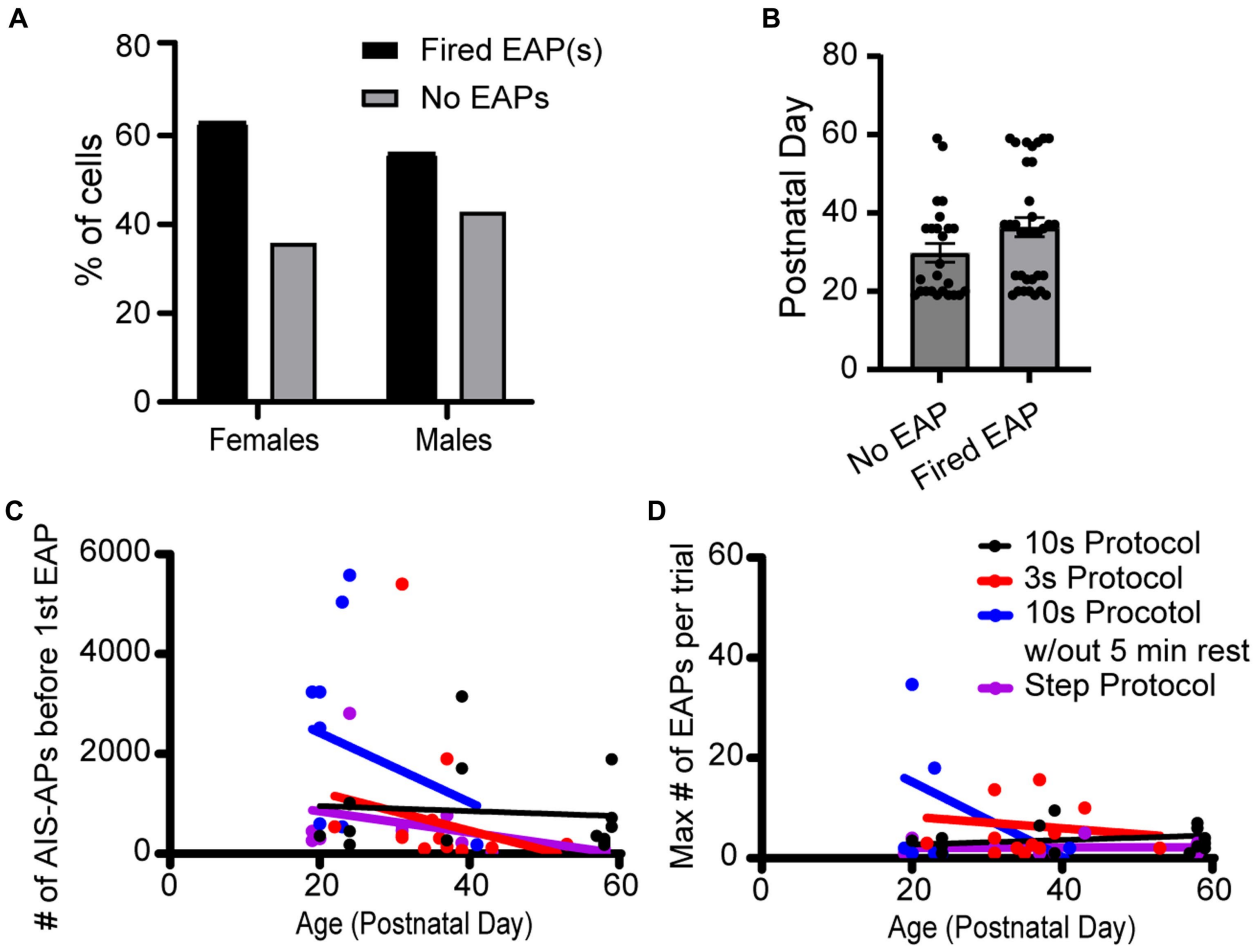
Sex and age did not appear to impact EAP firing in any protocols. **(A)** Data set across all protocols consists of 23 cells recorded from female and 43 cells recorded from male mice, of which 60.9 and 53.5% fired EAPs, respectively. There was no statistically significant difference between the percentage of cells that fired EAPs between females and males (*p* > 0.05; Chi-square test). **(B)** There was no statistically significant difference in animal age between cells that fired EAPs vs. cells that did not fire EAPs (*p* > 0.05, unpaired *t*-test). **(C)** For all the protocols, the total number of AIS-APs before the first EAP in relation to the mouse age in days. Dot color corresponds to the protocol the cell was recorded with. **(D)** Similarly, the maximum number of EAP(s) in relation to the mouse age in days. For both **(C,D)**, none of the simple linear regressions had a slope that was significantly deviated from zero.

The effect of animal sex and age on the probability to fire EAPs was not significant. Of the 23 cells from females, there was a 60.9% chance of eliciting EAPs whereas there was a 53.5% chance of eliciting EAPs in the 43 cells recorded in male mouse brain slices (Figures 7A,B). There was no statistically significant difference between the probability of males and females to fire EAPs (*p* = 0.310; Chi-square test). We next examined whether there was a correlation between the mouse age at the time of each experiment and EAP initiation threshold. Similar approaches were taken to examine the correlation between mouse age at the time of each experiment and EAP robustness. None of our linear regression analyses had a slope that significantly deviated from zero (Figures 7C,D).

## 3 Discussion

### 3.1 Prevalence of EAPs in RS cells

The canonical view of neuronal saltatory conduction is that it occurs in an anterograde direction along axons. However, recent data have indicated that axons have much wider-ranging functional capacity than previously thought (Debanne, 2004; Bucher and Goaillard, 2011). One such function involves backward propagation along the axon in the form of EAPs. EAPs have been described in nonpathological states including in neuropeptide Y-expressing interneurons of the hippocampus (Sheffield et al., 2011), neurogliaform and fast spiking neurons of the piriform cortex, somatosensory cortex, and hippocampus (Suzuki et al., 2014), parvalbumin neurons in the dentate gyrus (Elgueta et al., 2015), and acetylcholine releasing neurons in the striatum (Liu et al., 2022). With regards to other organisms, there has been extensive literature describing action potentials originating from distal axons in crustaceans involved in processing sensorimotor, motor, and circuit information (Meyrand et al., 1992; Bucher et al., 2003; Chao et al., 2005; Daur et al., 2009, 2019) and in motor control of leeches (Kristan et al., 2005). Here, we have described, for the first time, that the majority of regular spiking cells, the most prevalent neuronal type in the mammalian neocortex, also appears to be capable of generating these action potentials. By exploring EAPs in the context of cortical pyramidal cells, our research contributes to the understanding of the broader implications of EAPs and their potential significance in neuronal excitability.

Our experiments suggest that at least half of RS cells in the orbitofrontal cortex are capable of firing EAPs under nonpathological conditions *in vitro*. The most effective induction protocols—stimulus frequencies of 30, 60, or 100 Hz, with several seconds of rest between stimulus trains— evoked EAPs in 72.7% of recorded RS cells. If results from the orbitofrontal cortex can be generalized, a majority of neocortical RS cells may be capable of generating EAPs under specific induction conditions. The large majority of RS cells are pyramidal neurons (McCormick et al., 1985; Bean, 2007). Pyramidal cells are the principal cells of the neocortex, and they are critically involved in all aspects of cortical processing and output (Mountcastle, 1998; Hattox and Nelson, 2007; Mao et al., 2011). It is thus possible that EAPs generated by pyramidal cells play a widespread yet unrecognized role in neocortical function.

Our results demonstrate that RS cells are more likely to fire EAPs after firing AIS-APs at specific frequencies with a resting period between stimulus trials compared to previously used step protocols. The fixed AIS-APs number protocol (10 s) was effective at eliciting at least one EAP in 72.7% of recorded cells, while the fixed time interval protocol (3 s) and the 10 s protocol without rest were effective between 39.1 and 57.1% of the time, respectively. Given that allowing cells to recover between each stimulus train elicited EAPs in a higher proportion of RS cells, we hypothesize that certain mechanisms involved in EAP firing may become desensitized to incoming AIS-APs and require time to reset. Furthermore, our data demonstrate that EAP induction happens over the course of an average of 81.4 ± 57.7 s or 863.8 ± 1214.0 AIS-APs, suggesting that the mechanisms involved may have slow kinetics and have a (similarly slow) inactivation period prior to new EAP initiation. Together, these results imply that the mechanisms required to trigger ectopic firing are sensitive to the number and possibly frequency of AIS-APs, as well as have both a long integration period and slow recovery time. These findings have functional implications for neocortical RS cells: once a series of EAPs is generated, these cells require a period of relative quiescence to “reset” the EAP initiation mechanisms.

While we do not explore the mechanisms of EAP generation in neocortical pyramidal cells here, it has been shown that multiple manipulations that impact presynaptic terminal membrane potentials could be involved in EAP generation. Potential mechanisms include interactions between neurotransmitter transporters on astrocytes leading to local glutamate release and stimulation of presynaptic glutamate receptors (Deemyad et al., 2018), extracellular potassium accumulation (Keros and Hablitz, 2005), GABAA receptor stimulation (Avoli et al., 1998), extracellular fluctuations in calcium concentration, blockade of presynaptic voltage-gated potassium channels (Pinault, 1995), etc. Intrinsic membrane properties like a slow-down of repolarization at synaptic terminals and changes in genetic expression of membrane channels have also been proposed (Pinault, 1995).

Our recordings also indicate that EAPs can have varying amplitudes in somatic recordings not only across different cells, but within the same neuron. This is in-line with prior reports in the literature, and suggests that EAP amplitude is related to the extent to which a given EAP is able to propagate toward, and into the cell body. Failure of propagation at axon branch points may lead to low-amplitude EAPs, whereas failure to propagate at the AIS may lead to medium-amplitude EAPs. It is thus likely that smaller amplitude EAPs are generated more distally in the axon or dendrites (due to a higher probability of a branch-point failure), while larger amplitude EAPs originate closer to the soma (Spruston, 2008; Sheffield et al., 2011).

### 3.2 Implications for network-level processing

The fact that neocortical RS cells can generate EAPs has important implications for understanding both neuronal excitability and information processing within the cortex. Ectopic (axonal) spike initiation expands the classical view that action potentials are generally initiated in the AIS (Popovic et al., 2011; Kole and Stuart, 2012; Hu and Bean, 2018; Katz et al., 2018). Given that EAPs have been shown to, like their AIS-initiated counterparts, trigger synaptic transmission (unpublished observations), it is possible that they are an integral and under-explored part of normal network function.

During ectopic firing, a neuron generates action potentials in response to its own firing patterns over the preceding tens-of-seconds to minutes, and once it starts firing these EAPs it does so largely independently from that neuron’s inputs (Thome et al., 2018). This represents a fundamentally different process from the integration of voltage inputs at the axon hillock leading to AIS-AP initiation. While EAPs are being generated, the cells are likely much less sensitive to postsynaptic inputs than AIS-APs by virtue of where they are being generated, physically and electrotonically distal to the axon initial segment.

EAPs may enhance irregularity in the firing of single neurons by introducing a series of cell-generated action potentials during periods of coordinated network activity. Such irregular firing of single neurons has been shown, alongside interplay between excitatory and inhibitory cells, to support gamma rhythms (Buzsáki and Wang, 2012). Others have more directly suggested that EAPs play a role in the generation and/or maintenance of gamma oscillations (Dugladze et al., 2012; Thome et al., 2018), which are thought to play an important role in neocortical processing, specifically temporal coordination of cortical regions (Gray and Singer, 1989; Fries, 2009). Our data demonstrate that a majority of RS cells are capable of firing EAPs, and thus that, when engaged, they take part in gamma oscillations. Network states, including gamma oscillations, can cause neuronal activity that is similar in frequency and patterning to the stimulus paradigms we used to trigger ectopic activity. Sohal et al. (2009) demonstrated gamma oscillations in PV cells drive rhythmic firing at gamma frequency patterns (much like the stimulus paradigms we used to elicit EAPs) in downstream pyramidal cells. Since the stimulus frequencies we use in this study are in the gamma range, we hypothesize that during network conditions that include strong gamma frequency components it is likely that RS cells fire EAPs. Future *in vivo* studies will be necessary to determine whether this is the case. Another role that EAPs may play is more straightforward: since EAPs occur after periods of increased excitation it is possible that they may serve as a feed-forward amplification mechanism.

Our data show that some RS cells fire EAPs after they are induced by spiking at one of the frequencies we tested, while other RS cells are not as frequency-constrained. This suggests that certain network contexts will differentially evoke EAPs in subpopulations of RS cells, and raises the question of whether frequency dependence might be related to RS cell subtype. Our preliminary analysis of intrinsic properties (input resistance, AP threshold, and AP half-width) did not reveal any association between these properties and whether cells fired EAPs. We did not have a sufficient ‘n’ to determine whether the frequencies required to elicit EAPs in individual cells correlated with their intrinsic properties. Future work examining whether certain frequencies of firing elicit EAPs differentially across identified subtypes of pyramidal cells would be of great interest.

Our finding that some RS cells generate EAPs after firing in certain frequency ranges, while others are less selective suggests that there are two general classes of EAP firing in RS cells: (1) those that fire EAPs with less frequency specificity could serve to generally amplify network activity, and (2) RS cells that exhibit frequency-dependent EAP firing induction could serve as feed-forward amplifiers only when there is sufficient activity in a specific frequency range. Additionally, it is of interest to note that the spontaneous timing of EAPs after induction may contribute to the random components of neural activity in cortical circuits (Faisal et al., 2008; Yarom and Hounsgaard, 2011).

### 3.3 Implications for pathology

A better understanding of how EAPs arise during physiological conditions can help explain what leads to EAPs in pathological conditions. Studies have shown that EAP firing can occur in various epilepsy models (Rosen and Vastola, 1971; Gutnick and Prince, 1972; Stasheff and Wilson, 1990), suggesting that EAPs may be a contributing factor to the development and maintenance of epileptiform activity. Conversely, they could be acting to homeostatically prevent runaway excitation that could lead to a seizure when activated in interneurons. By studying the biophysical properties of EAPs, we can better understand whether, and how, they contribute to seizures, and may identify potential targets for therapeutic intervention along the way. Studies have shown that a wide range of pharmaceutical approaches can modulate EAPs, ranging from blocking ionotropic glutamate-receptor-mediated neurotransmission in hippocampal pyramidal neurons (Thome et al., 2018) to administration of neuromodulators such as acetylcholine or dopamine (Hoffman and Johnston, 1999).

Given the significance of EAPs in both physiological and pathological conditions, further investigation into both the role of, and mechanism underlying, EAPs in RS cells is warranted. Though our study focused on the orbitofrontal cortex, RS cells are found throughout the neocortex and may fire EAPs under different conditions depending on brain layer, local circuitry, and RS cell subtype. Teasing apart the exact mechanism behind EAPs in RS cells could help further our understanding of neural coding and signaling, both in physiological and pathological conditions, and in *in vitro* vs. *in vivo* conditions.

## 4 Methods

### 4.1 Mice

Mice were obtained from the Brown University Animal Care Facility (ACF). All procedures were approved by, and complied with all ethical regulations of, the Brown University Institutional Animal Care and Use Committee (IACUC). The following mouse lines were used: PV-Cre (The Jackson Laboratory, 008069), GFAP-ChR (The Jackson Laboratory, 024098), NTSR1-Cre (received from C.I. Moore, Brown University, generated by the GENSAT project, available at the Mutant Mouse Regional Resource Centers [MMRRC], 030648-UCD), Ai32 (The Jackson Laboratory, 012569), Ai14 (The Jackson Laboratory, 007908), ICR (Charles River, CD-1[ICR], Strain Code 022). All mice used in this study had ICR genetic backgrounds. Mice were maintained on a 12 h:12 h light/dark cycle, group-housed, and provided food and water *ad libitum*.

### 4.2 Dissection, slice preparation, and solutions

Brain slices were prepared from P18-P60 mice of either sex (randomly chosen) for *in vitro* recordings using both hemispheres in the sagittal plane, as previously described (Crandall et al., 2017; Martinez-Garcia et al., 2020). The mice were deeply anesthetized with isofluorane, then decapitated. The brains were carefully dissected out of the skull after removing the fur and cutting the skull down the midline, above the olfactory bulbs, and below the brain with dissection scissors (Roboz, RS 5910). The skull was then peeled away with forceps and the cerebellum was removed with a scalpel. The brain was then immediately submerged in cold (4°C), oxygenated slicing solution containing (in mM): 3 KCl, 1.25 NaH2PO4, 10 MgSO4, 0.5 CaCl2, 26 NaHCO3, 10 glucose, and 234 sucrose. The brain hemispheres were mounted, using a cyanoacrylate adhesive, onto the stage of a vibrating tissue slicer (VT 1200S, Leica Microsystems, Germany). 300 μm thick parasagittal brain slices containing the orbitofrontal cortex were obtained, while maintaining the olfactory bulb, which served as both an anatomical landmark and a convenient surface for one of two platinum weights we used to hold the slice in place during recordings. Slices were incubated in a holding chamber at 32°C for 15 min following slicing, and left in the same chamber at room temperature for 45 min prior to recording. Throughout the experiment day, the slices were maintained in the holding chamber, which contained oxygenated (5% CO2, 95% O2) holding solution containing (in mM): 126 NaCl, 3 KCl, 1.25 NaH2PO4, 2 MgSO4, 1.2 CaCl2, 26 NaHCO3, and 10 glucose.

### 4.3 Whole-cell recording procedure

Brain slices (300 μm) were placed in a submersion-type recording chamber maintained at 30 ± 3°C and continuously perfused with oxygenated (5% CO2, 95% O2) artificial cerebrospinal fluid (ACSF) solution, which consisted of (in mM): 126 NaCl, 3 KCl, 1.25 NaH2PO4, 1 MgSO4, 1.2 CaCl2, 26 NaHCO3, and 10 glucose. Neurons were visualized for recording using infrared light and differential interference contrast optics with low (4X) and high (40X water immersion) objectives on a BX50WI (Olympus Corporation, Tokyo, Japan) series microscope with a high sensitivity digital camera (Hamamatsu Orca BT Fusion S, Hamamatsu Photonics, Japan) and HC Image software (Hamamatsu Photonics). Only neurons at least three cell layers deep were recorded to maximize cell health and integrity. Patch pipettes with tip resistances of (4.5–7 MΩ) were filled with a potassium gluconate-based internal recording solution containing (in mM): 130K-gluconate, 4 KCl, 2 NaCl, 10 HEPES, 0.2 EGTA, 4 ATP-Mg, 0.3 guanosine triphosphate (GTP)-Tris, and 14 phosphocreatine-K (pH 7.25, ∼290 mOsm). During all recordings, pipette capacitances were neutralized. Series resistances were between (~5–35 MΩ) and were compensated for.

### 4.4 Recording protocols

We identified Regular Spiking (RS) cells in the orbitofrontal cortex by their firing pattern in response to 600 ms long current steps, starting at −25 pA, and increasing by 5 pA every 3 s (the “step” protocol).

The resting membrane potential of the cells was monitored during intertrial periods using the ‘gap free’ protocol, during which no current pulses were injected. This was done to allow for recovery of ectopic firing, and to evaluate cell health and monitor for any ongoing ectopic firing between trials. We allowed 5 min for cells to recover between current injections (step protocol or frequency stimulations), except where otherwise specified. Note that all voltages reported are not corrected for the liquid junction potential, which we have measured as 12.5 mV.

To evoke EAPs, we utilized multiple current injection patterns in the whole-cell patch clamp configuration (in current clamp). These patterns are divided into three main approaches: the step protocol, frequency stimulations with a fixed number of AIS-APs elicited over a longer, variable time interval and frequency stimulations with AIS-APs elicited at varying frequencies over a shorter, pre-specified time interval. The fixed AIS-AP number stimulations consisted of 180 high amplitude current pulses delivered at 30, 60, or 100 Hz over 10 s trials. The fixed time interval stimulations consisted of 1 s of high amplitude current pulses delivered at 30, 60, or 100 Hz over 3 s trials: at 30 Hz stimulation frequency, 30 current pulses were delivered over 1 s in each trial; at 60 Hz stimulation frequency, 60 current pulses were delivered over 1 s in each trial; at 100 Hz stimulation frequency, 100 current pulses were delivered over 1 s in each trial. Amplitudes of each current pulse started at 2500 pA and were titrated on a cell-by-cell basis until cells fired an AIS-AP for at least 80% of current pulses. Step protocols were stopped after the cell reached depolarization block. Frequency stimulations with fixed numbers of AIS-APs (10 s and 10 s without rest protocols) were stopped after the cell fired 4,000 AIS-APs. Frequency stimulations with fixed time intervals (3 s protocols) were stopped after 10,000 AIS-APs.

Data were collected utilizing a Multiclamp 700B (Molecular Devices, San Jose, CA) amplifier, and digitized with a Digidata 1,440 (Molecular Devices). We interfaced with the digitizer and amplifier using Multiclamp Commander and pClamp version 10 (Molecular Devices). Signals were low-pass filtered at 10 kHz and were digitized at 20 kHz.

### 4.5 Biocytin filling of RS cells

For select cells (*n* = 5), biocytin was added to the internal solution at a final concentration of 0.25%. After break-in the cell was maintained for at least 5 min to allow diffusion of biocytin into the cell. After detaching the pipette, slices were maintained in the recording chamber for 5 min, then placed in 4% paraformaldehyde for 30 min at room temperature. Slices were then washed five times in 0.1 M phosphate buffer containing 0.15 M NaCl, pH 7.4 (PBS) at room temperature (5 min per wash), transferred to 30% sucrose/PBS, and kept at 4°C overnight. Slices were again washed five times in PBS at room temperature, then blocked in 10% blocking solution (normal goat serum diluted in 0.2% Triton X-100 in PBS for 2 h at room temperature). Slices were washed five times in PBS at room temperature, incubated for 2 h in streptavidin-Alexa488 conjugate (concentration: 1:1000, in 1% blocking solution), then washed again five times in PBS at room temperature. Slices were mounted in VECTASHIELD aqueous mounting medium (Newark, California) and subsequently visualized on an Olympus FV3000 confocal microscope.

## Data availability statement

The raw data supporting the conclusions of this article will be made available by the authors, without undue reservation.

## Ethics statement

The animal study was approved by Brown University Institutional Animal Care and Use Committee. The study was conducted in accordance with the local legislation and institutional requirements.

## Author contributions

YZ: Writing – original draft, Writing – review & editing, Conceptualization, Data curation, Formal analysis, Investigation, Methodology, Software, Validation, Visualization. SS: Writing – original draft, Writing – review & editing, Conceptualization, Data curation, Formal analysis, Investigation, Methodology, Validation, Visualization. AL: Writing – original draft, Data curation, Visualization. SD: Conceptualization, Methodology, Writing – original draft. BC: Funding acquisition, Project administration, Resources, Supervision, Writing – review & editing. BT: Conceptualization, Funding acquisition, Methodology, Project administration, Resources, Supervision, Software, Writing – review & editing.
